# Cardioprotection via Metabolism for Rat Heart Preservation Using the High-Pressure Gaseous Mixture of Carbon Monoxide and Oxygen

**DOI:** 10.3390/ijms21228858

**Published:** 2020-11-23

**Authors:** Chiharu Suzuki, Naoyuki Hatayama, Tadashi Ogawa, Eri Nanizawa, Shun Otsuka, Koichiro Hata, Hiroshi Seno, Munekazu Naito, Shuichi Hirai

**Affiliations:** 1 Department of Anatomy, Aichi Medical University, Aichi 480-1195, Japan; chihalily39@gmail.com (C.S.); nanizawa.e@aichi-med-u.ac.jp (E.N.); ootsuka.shun.889@mail.aichi-med-u.ac.jp (S.O.); munekazunaito@gmail.com (M.N.); shinamon611@gmail.com (S.H.); 2 Department of Legal Medicine, Aichi Medical University, Aichi 480-1195, Japan; ogawatd@aichi-med-u.ac.jp (T.O.); senoyo@aichi-med-u.ac.jp (H.S.); 3 Department of Surgery, Division of Hepato-Biliary-Pancreatic and Transplant Surgery, Graduate School of Medicine, Kyoto University, Kyoto 606-8507, Japan; khata@kuhp.kyoto-u.ac.jp

**Keywords:** cardioprotection, ischemic injury, metabolomics, carbon monoxide, oxygen, high-pressure gas, transplantation, rat

## Abstract

The high-pressure gas (HPG) method with carbon monoxide (CO) and oxygen (O_2_) mixture maintains the preserved rat heart function. The metabolites of rat hearts preserved using the HPG method (HPG group) and cold storage (CS) method (CS group) by immersion in a stock solution for 24 h were assessed to confirm CO and O_2_ effects. Lactic acid was significantly lower and citric acid was significantly higher in the HPG group than in the CS group. Moreover, adenosine triphosphate (ATP) levels as well as some pentose phosphate pathway (PPP) metabolites and reduced nicotinamide adenine dinucleotide phosphate (NADPH) were significantly higher in the HPG group than in the CS group. Additionally, reduced glutathione (GSH), which protects cells from oxidative stress, was also significantly higher in the HPG group than in the CS group. These results indicated that each gas, CO and O_2_, induced the shift from anaerobic to aerobic metabolism, maintaining the energy of ischemic preserved organs, shifting the glucose utilization from glycolysis toward PPP, and reducing oxidative stress. Both CO and O_2_ in the HPG method have important effects on the ATP supply and decrease oxidative stress for preventing ischemic injury. The HPG method may be useful for clinical application.

## 1. Introduction

Transplantation is an important treatment approach for end-stage diseases. As the organ must be removed from the body and preserved for a certain period of time, an ischemic injury is an inevitable event during the organ transplant procedure. Currently, the preservation time for clinically extracted hearts before transplantation is 4–6 h [1]. To enhance the current quality of donor hearts, preservation methods have been continuously developed. Although the improvement of preservation solutions has made it possible to maintain the nutritional supply and electrolyte balance, the critical method to supply oxygen (O_2_) is difficult and has not yet been established. Therefore, injury caused by exposure to hypoxia from ischemia has been one of the major problems in organ preservation.

The heart requires adenosine triphosphate (ATP) to maintain contractile function and rapidly and continuously resynthesizes ATP within cardiomyocytes. The heart contains approximately 30–40% mitochondria within the cardiomyocyte volume, making its demands for O_2_ higher than those of other organs [2]. O_2_ concentration has been found to be closely related to increased oxidative stress by altering the responsible cytochrome chain activity [3]. Hypoxia in ischemia induced ATP synthesis and increased reactive oxygen species (ROS) while decreasing the activity of the normal cellular antioxidant system [4]. The resulting oxidative stress in the organ transplant procedure initiates cellular injury [5]. The balance between oxidative and antioxidant systems is one of the important factors for maintaining the desired condition of the preserved organ and achieving success in transplantation.

Carbon monoxide (CO) is regarded as poisonous because of its high affinity for hemoglobin that rapidly increases carboxyhemoglobin to toxic levels that compromise O_2_ delivery to the tissues. On the contrary, CO is constantly produced in the body and exerts vasoactive, antiproliferative, antioxidant, anti-inflammatory, and antiapoptotic effects and significantly contributes to cell protection, including the heart, and has been assessed for its cytoprotective and cytotherapeutic properties [6]. A previous report demonstrated that a high-pressure gas (HPG) preservation method using a mixture of CO and O_2_ gases successfully resuscitated extracted rat hearts after 48 h of preservation [7]. Rat hearts exposed to hyperbaric CO and O_2_ without immersion in cold storage (CS) solution has been found to effectively obtain necessary cell-protective effects of gas for preservation [8]. The rat heart function after 24 h of HPG preservation using a mixture of CO and O_2_ gases was also found to be almost the same as that in control hearts that were transplanted immediately after extraction [9]. It was expected that these gases might induce ATP supply for reducing oxidative stress due to ischemia. Therefore, this study focused on metabolites to establish the effects of CO and O_2_ gases for cardioprotection during rat heart preservation and analyzed factors associated with the improvement of the HPG method with CO and O_2_ gases.

## 2. Results

### 2.1. Comparison of Hearts with Heterotopic Cervical Transplantation after 24-h Preservation under Cold Storage and a High-Pressure Gaseous Mixture of Carbon Monoxide and Oxygen

**Figure 1:**
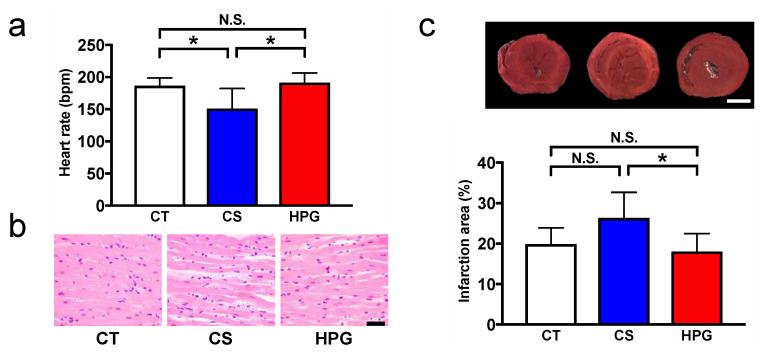
Assessment of transplanted hearts after 24-h preservation in three groups: control group (CT), nonpreservation; cold storage (CS) group, preservation in the University of Wisconsin (UW) solution for 24 h; high-pressure gas (HPG) group, preservation using the mixture of high-pressure carbon monoxide (CO) and oxygen (O_2_) for 24 h. (**a**) Heart rate at 60 min after transplantation. (**b**) Hematoxylin and eosin staining in transplanted hearts after 24-h preservation. Black bar represents 40 μm. (**c**) Representative midmyocardial cross-sections of 2,3,5-triphenyltetrazolium chloride-stained hearts. Red-stained areas indicate viable tissues, and white areas indicate infarct tissues. White bar represents 500 μm. Myocardial infarction areas in the hearts of each group were quantified. Data in each bar are presented as mean ± standard error of the mean. N.S., not significant. * *p* < 0.05.

The heart beat rates of transplanted hearts of each group are shown in Figure 1a.

After heterotopic cervical heart transplantation, the heart beat rates of the CS group were significantly reduced compared with the control group (CT) and the HPG group (151.1 ± 31.26 bpm vs. 186.3 ± 12.38 bpm vs. 191.3 ± 14.67 bpm). No significant difference was observed between the CT and HPG groups (Figure 1a). Light microscopy evaluation revealed that the myocardium in the HPG group maintained an almost normal tissue structure and shape, with the myocardial fibers arranged in an orderly pattern. However, the myocardium in the CS group showed erythrodiapedes between some muscle fibers (Figure 1b). The myocardial infarct area that was evaluated using 2,3,5-triphenyltetrazolium chloride (TTC) staining as an indicator of mitochondrial activities was larger in the CS group (26.3% ± 3.99%) than in the CT (19.8% ± 3.51%) and HPG (18.0% ± 3.31%) groups (Figure 1c).

### 2.2. Comparison of Cardiac Morphology and Mitochondrial Activities after 24-h Preservation under Cold Storage and High-Pressure Gaseous Mixture of Carbon Monoxide and Oxygen

**Figure 2:**
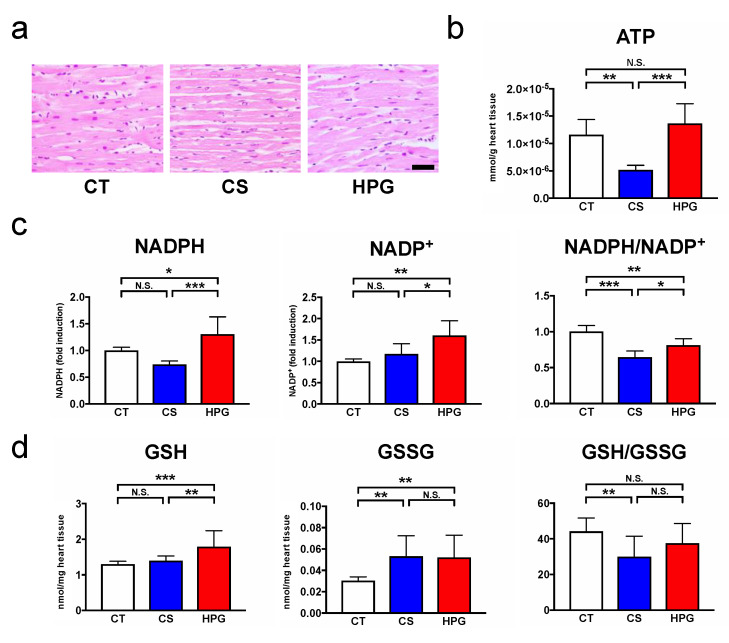
Assessment of cardiac morphology and mitochondrial activities after 24-h preservation. (**a**) Hematoxylin and eosin staining in hearts immediately after 24-h preservation. Black bar represents 40 μm. (**b**) Adenosine triphosphate (ATP) content. (**c**) Alterations in reduced nicotinamide adenine dinucleotide phosphate (NADPH), NADP^+^, and NADPH/NADP^+^ ratio. Bar graphs indicate fold changes relative to the CT group. (**d**) Alterations in reduced glutathione (GSH), oxidized glutathione (GSSG), and the GSH/GSSG ratio. Data in each bar are presented as mean ± standard error of the mean. N.S., not significant. * *p* < 0.05, ** *p* < 0.01, *** *p* < 0.001.

Light microscopy revealed that the myocardium in the CS and HPG groups maintained an almost normal tissue structure and shape (Figure 2a). However, ATP-producing abilities in the CS group were significantly lower than those in the HPG group (Figure 2b). Reduced nicotinamide adenine dinucleotide phosphate (NADPH), which is produced in the pentose phosphate pathway (PPP), and reduced glutathione (GSH), which is generated by reducing oxidized glutathione (GSSG) using NADPH, were significantly higher in the HPG group than those in the CS group (Figure 2c,d). Moreover, the ratio of GSH to GSSG, which indicates the condition of cellular oxidative stress, was significantly decreased in the CS group, but not in the HPG group, compared with the CT group (Figure 2d).

### 2.3. Selection of Important Differential Metabolites

**Figure 3:**
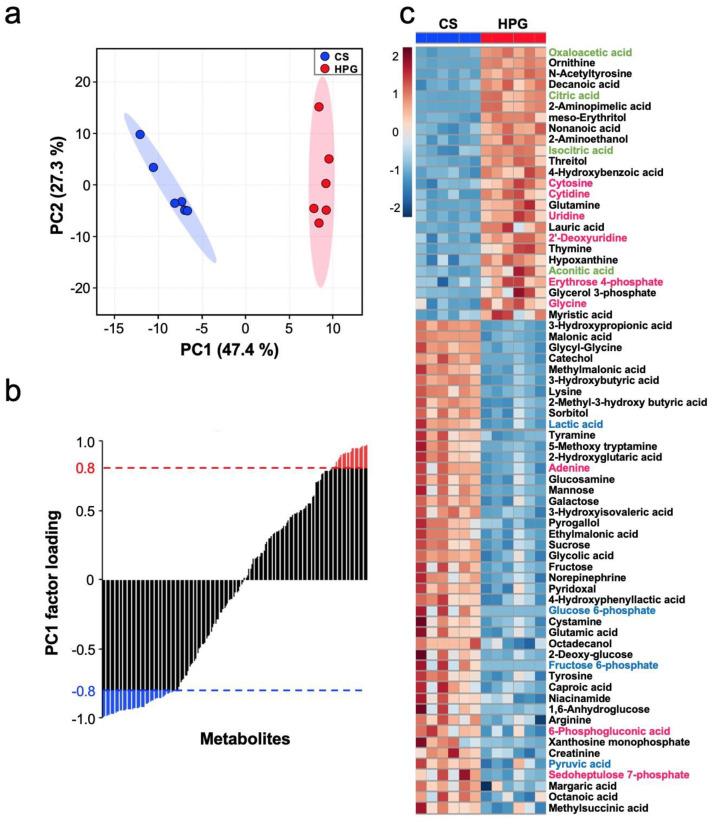
Metabolite profiling of hearts preserved using the cold storage (CS) method and high-pressure gas (HPG) method. (**a**) Principal components analysis (PCA) score plot. Blue and red circles represent 95% confidence interval of CS and HPG, respectively. (**b**) Factor loading plot for PC1. A total of 70 statistically significant metabolites were selected with an absolute value of 0.8 of PC1 factor loading. Blue and red dotted lines show the significantly negative and positive levels, respectively. (**c**) A heat map of 70 statistically significant metabolites in the CS and HPG groups. Red, green, and blue letters show metabolites of the pentose phosphate pathway and nucleic acid, tricarboxylic acid (TCA) cycle, and glycolysis, respectively. The color scale from blue to red indicates low to high amount of metabolites between CS and HPG.

Six heart samples each of the CT, CS, and HPG groups were used for the metabolomic analysis by gas chromatography/tandem mass spectrometry (GC–MS/MS), and 172 metabolites were detected. A principal component analysis (PCA) of these metabolomic data was performed after the preprocessing of autoscaling (zero mean and divided by the standard deviation). The PCA score plot indicated that the CT, CS, and HPG groups were completely different (Supplementary Figure S1). The PCA score plot between the CS and HPG groups using the CO and O_2_ mixture showed that the PC1 scores of the CS and HPG were negative and positive, respectively (Figure 3a). The sum of PC1 and PC2 variances was explained at almost 75%. This result suggested the PC1 score is positively related to the effect of CO or O_2_ gases in HPG preservation. Metabolites are sorted in ascending order of the value for PC1 factor loading, and 70 statistically significant metabolites were selected with an absolute value 0.8 of PC1 factor loading: 45 significantly negative metabolites and 25 significantly positive metabolites (Figure 3b).

### 2.4. Alternations in Metabolic Pathway

**Figure 4:**
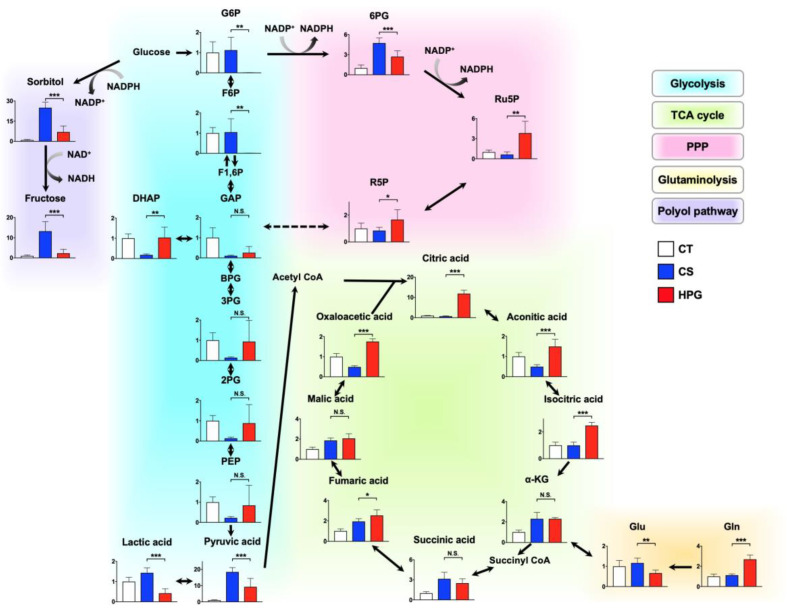
Metabolome data map after 24-h heart preservation in glycolysis, tricarboxylic acid (TCA) cycle, pentose phosphate pathway (PPP), glutaminolysis, and polyol pathway. Bar graphs indicate fold changes relative to the control (CT) group. The lack of a bar graph representation for a given metabolite means that the metabolite was not detected. Data in each bar are presented as mean ± standard error of the mean. N.S., not significant. * *p* < 0.05, ** *p* < 0.01, *** *p* < 0.001. CS, cold storage; HPG, high-pressure gas; G6P, glucose 6-phosphate; F6P, fructose-6-phosphate; F1,6P, fructose 1,6-bisphosphate; GAP, glyceraldehyde 3-phosphate; DHAP, dihydroxyacetone phosphate; BPG, bisphosphoglycerate; 3-PG, 3-phosphoglycerate; 2-PG, 2-phosphoglycerate; PEP, phosphoenolpyruvate; α-KG, α-ketoglutarate; 6PG, 6-phosphogluconate; Ru5P, ribulose-5-phosphate; R5P, ribose-5-phosphate; Glu, glutamic acid; Gln, glutamine.

The heat map of 70 statistically significant metabolites revealed that 25 significantly positive metabolites contained six metabolites belonging to PPP and nucleic acid and four metabolites belonging to tricarboxylic acid (TCA) cycle, suggesting that the PPP and TCA cycle tended to increase in the HPG group (Figure 3c). A detailed comparative analysis of metabolite changes of the CS and HPG groups revealed that ribulose-5-phosphate (Ru5P) and ribose-5-phosphate (R5P) of the HPG group in PPP were significantly higher than those of the CS group, whereas 6 PG in PPP of the HPG group was significantly lower than that of the CS group. Moreover, citric acid, aconitic acid, isocitric acid, fumaric acid, and oxaloacetic acid in the TCA cycle related to aerobic metabolism were significantly higher in the HPG group than in the CS group. Moreover, in glutaminolysis, glutamate was significantly higher in the CS group, whereas glutamine was significantly higher in the HPG group (Figure 4). On the contrary, metabolites belonging to anaerobic glycolysis tended to be high in the CS group (Figure 3c). A detailed comparative analysis on changes in the metabolites of the CS and HPG groups revealed that glucose 6-phosphate (G6P) and fructose-6-phosphate (F6P) in glycolysis, and lactic acid, which is a by-product of anaerobic metabolism, were higher in the CS group than in the HPG group. Furthermore, sorbitol and fructose of the CS group in the polyol pathway were significantly higher than those of the HPG group (Figure 4).

## 3. Discussion

**Figure 5:**
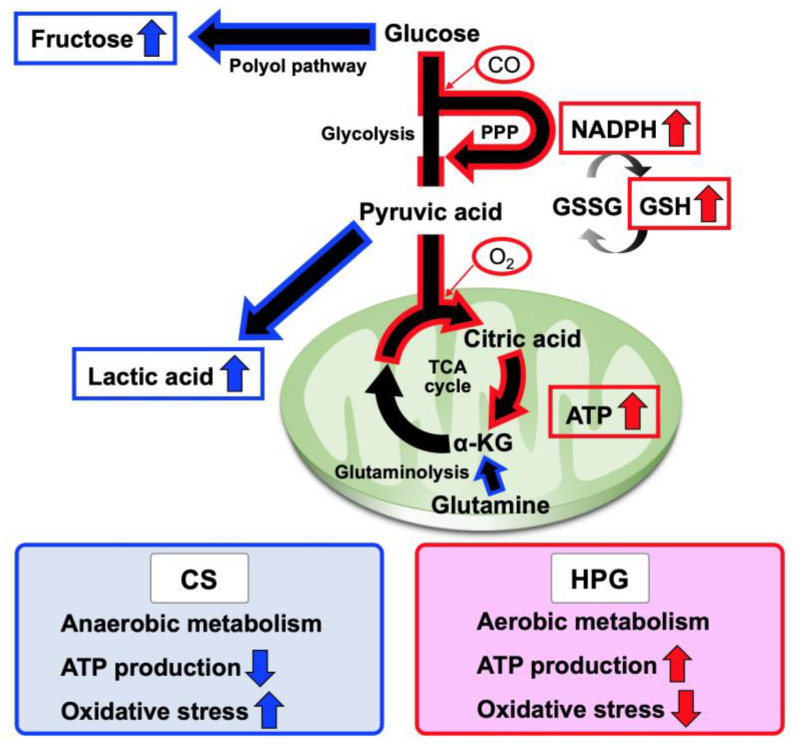
Schematic representation of proposed metabolic pathways for the preservation in comparison between the cold storage (CS) method and high-pressure gas (HPG) method. The CS preservation method is characterized by lactic acid production, a by-product of anaerobic metabolism, the polyol pathway that causes oxidative stress, and anaplerotic utilization of glutaminolysis. Conversely, the HPG preservation method is characterized by adenosine triphosphate (ATP) production and a shift in glucose metabolism toward the pentose phosphate pathway (PPP). PPP acceleration increases cellular reduced nicotinamide adenine dinucleotide phosphate (NADPH) amounts, providing reduced glutathione (GSH) that benefits reducing oxidative stress.

The heart functions and mitochondrial activities in rat hearts are better maintained using the HPG method, which includes a mixture of CO and O_2_, than using the CS method. A total of 70 statistically significant metabolites were detected in the preserved hearts between the HPG and CS methods (Figure 3b,c). With respect to metabolites, preserved hearts in the HPG group have been suggested to activate the TCA cycle and PPP, whereas anaerobic metabolisms were induced in those of the CS group. Consequently, ATP production was significantly reduced in the CS group but not in the HPG group due to TCA activation. Furthermore, the PPP activation may have induced the increase in NADPH and GSH, with the result that GSH/GSSG ratio related to oxidative stress did not significantly decrease in the HPG group (Figure 2 and Figure 4). Both CO and O_2_ in the HPG method have been suggested to have important effects on the ATP supply and decrease oxidative stress through the activation of the PPP and TCA cycle (Figure 5).

During preservation, donor organs are exposed to significant periods of hypoxia in the CS method. The lack of O_2_ during preservation is detrimental to cellular viability and damage on ischemia–reperfusion injury. Hypoxia induces glycogen breakdown by anaerobic glycolysis, producing two molecules of ATP along with lactic acid [10,11]. Excessive anaerobic metabolism and accumulation of lactic acid result in decreased tissue pH, which then acts through negative feedback to inhibit further ATP production [12]. In this study, ATP levels were found to be significantly decreased in the preserved rat hearts in the CS group, but not in the HPG group, compared with the CT group (Figure 2b). Due to ATP deficiency, the hexokinase activity on glycolysis was suppressed, and the excess glucose entered the polyol pathway when aldose reductase reduced it to sorbitol. This reaction oxidized NADPH to NADP^+^ and increased oxidative stress [13,14]. This study indicated that sorbitol and fructose in the polyol pathway of the CS group were significantly higher than those of the HPG group. Moreover, it is believed that the decreased NADPH/NADP^+^ ratio was one of the causes of oxidative stress, resulting in cell damage in the CS group. Moreover, citric acid might be supplied via glycolysis, but not glutaminolysis, and then regenerated by the TCA cycle, which was significantly increased in the HPG group (Figure 4). It was demonstrated that ATP in the preserved heart of the HPG group was maintained accordingly. These results suggested that O_2_ supply was adequate, and that aerobic metabolism was functioning in the HPG group. The development of the O_2_ supply method has been a major research focus in the field of organ preservation [15,16]. Organ oxygenation has been demonstrated to be inadequate even if the organ preservation solution around the organ is fully saturated with 100% O_2_ [17]. The study showed the possibility that the HPG method directly supplies O_2_ to the organ from the outside in the gas state, thus maintaining the mitochondrial aerobic function and preventing ischemic injury during organ preservation.

Recently, CO has been reported to regulate phosphofructokinase/fructose bisphosphatase type-3 methylation and determine glucose utilization [18]. Moreover, CO accelerated PPP, increasing the NADPH concentration and decreasing the ratio of reduced to oxidized glutathione (GSH/GSSG). As a result, CO improved resistance against oxidative stress for hypoxia due to ischemia [19]. The rat hearts in the CS group were ischemic and exhibited significantly decreased GSH/GSSG ratio by oxidation. Conversely, the ratio in the HPG group was not significantly lower than that in the CT group (before preservation) (Figure 2d). Moreover, NADPH in the HPG group was significantly higher than that in the CT and CS groups (Figure 2c). On the analysis of GC–MS/MS, Ru5P and R5P suggested that PPP was accelerated in the HPG group (Figure 4). In this way, including CO in the HPG method was thought to be effective in reducing oxidative stress during rat heart preservation (Figure 5).

Ischemia–reperfusion injury is at the center of the pathology of the most common cardiovascular diseases and can affect mitochondrial function. Mitochondrial dysfunction mostly affects myocardial contractility but also predisposes one to arrhythmias due to the myocardium having high metabolic demand and mitochondrial content [20,21]. Although no major changes were observed in the myocardial structure at pretransplantation among the three groups (Figure 2a), after transplantation, erythrodiapedes was observed between some muscle fibers in the myocardium of the CS groups, whereas the myocardium of the HPG group was almost normal in structure (Figure 1b). Moreover, according to results of the heart rate (HR) and TTC staining, no significant differences were noted between the HPG and CT groups, but the results of the CS group were lower (HR) and higher (TTC: infarction area) than those of the CT group (Figure 1a,c, Supplementary Movie S1). Hypoxia-induced imbalance in the ATP supply has been reported to cause cell damage during ischemia–reperfusion [22]. This study demonstrated that the HPG preservation method could maintain mitochondrial aerobic function not only during preservation but also during reperfusion, based on the results of TTC staining. Conversely, inappropriate amounts of O_2_-induced oxidative stress have been reported [23]. Our previous reports also showed that rat hearts preserved in the HPG method with CO and O_2_ maintained better functions after the heterotopic cervical heart transplantation than those preserved in only CO or O_2_ gas, and partial pressures of O_2_ and CO also affected the posttransplant status. In this study, it was shown that CO might induce PPP and reduce oxidative stress. Therefore, there seems to be a better combination and partial pressure of gases for the HPG preservation method for the reduction of oxidative stress and maintaining mitochondrial aerobic function during preservation, resulting in reduced ischemia–reperfusion injury. Therefore, the detailed mechanism should be elucidated, and a better preservation method should be developed in the future.

This study has potential limitations. The HPG method using the CO and O_2_ mixture was compared only with the simple immersion method. The HPG method could be affected by various changes during storage in the gas phase, and the effects of only the CO and O_2_ combination were assessed. Therefore, various conditions such as oxygen only and carbon monoxide only should be assessed to clarify the effect of each gas.

In conclusion, this study suggested that the HPG preservation method with the CO and O_2_ mixture maintains the ischemic preserved organs in better condition by inducing ATP maintenance and oxidative stress reduction through the alteration of metabolic pathways.

## 4. Materials and Methods

### 4.1. Animals

An inbred line of LEW/SsN Slc rats (male, 10 weeks old; average weight, 230 g; range, 220–245 g; intact, *n* = 46; donors, *n* = 18; recipients, *n* = 18) was purchased from the Shizuoka Laboratory Animal Center (Shizuoka, Japan). Handling and care of the rats conformed to the National Institutes of Health guidelines for animal research, and all experimental protocols involving animals were approved by the Committee for Animal Care at Aichi Medical University (Permit No. 2019-54, 2020-57). All experiments involving animals were performed in accordance with relevant guidelines and experimental protocols. Every effort was made to minimize animal suffering.

### 4.2. HPG Preservation Method and Experimental Design

**Figure 6:**
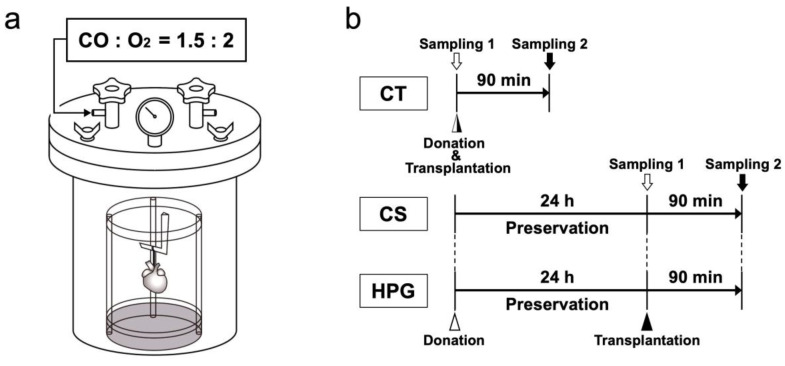
Schematic representation of the preservation method and experimental design. (**a**) The pressure tight chamber was filled with CO [partial pressure of carbon monoxide (PCO) = 1500 hPa] and O_2_ [partial pressure of oxygen (PO_2_) = 2000 hPa]. During heart preservation, a flask with 50 mL distilled water was placed in the chamber to maintain humidity. (**b**) Three groups were examined: nonpreserved hearts (CT group), hearts preserved in University of Wisconsin (UW) solution for 24 h (CS group), and hearts preserved with the high-pressure gas preservation containing CO and O_2_ for 24 h (HPG group). To assess mitochondrial activities and metabolomic analysis, the hearts were sampled immediately after heart extraction in the CT group and after preservation in the CS and HPG groups (Sampling 1: white arrow). For morphological and functional assessment, the hearts were sampled after 90 min from transplantation in the CS and HPG groups (Sampling 2: black arrow).

For the HPG preservation method, a chamber that could withstand high pressure was developed, as previously described (Nakamura Iron Works Co., Ltd. Tokyo, Japan) [8]. To prepare for organ preservation, the inne chamber was placed in distilled water to maintain humidity and then cooled to 4 °C. Extracted rat hearts suspended in a plastic cylinder were placed in the chamber, and the lid was closed using four bolts. The chamber was filled with CO [partial pressure of carbon monoxide (PCO) = 1500 hPa] and O_2_ [partial pressure of oxygen (PO_2_) = 2000 hPa]. During the heart preservation, a flask with 50 mL of distilled H_2_O was placed within the chamber to maintain humidity for 24 h. The inner chamber gas temperature was initially increased temporarily (peak 16.6 °C ± 0.8 °C), and the temperature was then decreased over approximately 80 s by placing the chamber in a fridge at 4 °C. Using this temperature alteration, the inner cardiac cavity temperature was increased by approximately 1.98 °C ± 0.53 °C. After the preservation, the preserved heart was removed, and gases were released from the chamber (Figure 6a).

The hearts were extracted from the rats under deep anesthesia using pentobarbital (50 mg/kg, Kyoritsu Seiyaku Corporation, Tokyo, Japan). Then, the blood was removed using Krebs–Henseleit solution following an aortic/pulmonary artery incision, and University of Wisconsin (UW) solution (Viaspan, Du Pont, Wilmington, DE, USA) was further infused. A custom-built seven air pressure-resistant chamber (L: 165 mm/W: 165 mm/H: 200 mm, material: iron, Nakamura Iron Works Co., Ltd. Tokyo, Japan) was cooled to 4 °C in advance. Next, the rat hearts were hung inside a chamber to preserve in the chamber at 4 °C under filling with a mixture of CO and O_2_ gases (HPG group, *n* = 6). Moreover, the hearts were completely immersed in the UW solution and then placed in a chamber filled with room air (1000 hPa) for 24 h (CS group, *n* = 6). The hearts used immediately after extraction without preservation were included in the CT group (*n* = 6). The preserved hearts were removed from the chamber to assess metabolites, as shown as sampling 1 in Figure 6b.

Heterotopic cervical heart transplantation to a recipient rat was performed for morphological and functional analyses, as described previously [8]. At 60 min following the heterotopic cervical heart transplantation, the heartbeat was counted. Transplanted hearts were sampled after 90 min from transplantation (CT, CS, and HPG groups, *n* = 6 for each group) as shown as sampling 2 in Figure 6b.

### 4.3. Light Microscopy

Both preserved and transplanted hearts from each group were fixed in 10% formalin. The samples were then washed, dehydrated with an ethanol series, and embedded in paraffin. Serial 6-μm sections were cut with a microtome and stained with Gill’s hematoxylin III and 2% eosin Y.

### 4.4. Mitochondrial Activity Estimation

The mitochondrial activity was estimated by TTC staining. Briefly, after the reperfusion, the hearts were cut into 2-mm thick slices vertical to the atrioventricular groove. The slices were stained by incubation in 2% TTC solution in the phosphate buffer (0.1 M) at 37 °C for 15 min and then fixed in 4% paraformaldehyde solution. Slices unstained by TTC were measured by planimetry using the Image Pro Plus 5.0 software (Media Cybernetics, Silver Spring, MD, USA) and expressed as a percentage of the total heart.

### 4.5. ATP Luciferase Assay

ATP levels in cardiac tissues were measured using the “Tissue” ATP assay kit (Toyo Ink group, Tokyo, Japan). Briefly, the tissue pieces (50 mg) were homogenized with lysis solution. The homogenized tissue was centrifuged at 1000× *g* at 4 °C for 10 min. The supernatant was mixed with ATP extraction buffer for 30 min at room temperature. The sample was mixed with luciferase reagent containing luciferin. Then, luciferase activity was measured using a luminometer (SpectraMax, Molecular Devices, Wuxi, China). The amount of ATP in each sample was calculated using the standard curve. Data were presented as relative abundance per gram of heart tissue.

### 4.6. NADP^+^ and NADPH Assay

NADP^+^ and NADPH in the cardiac tissues were measured using the NADP/NADPH-Glo™ Assay (Promega, Madison, WI, USA) according to the manufacturer’s protocol. NADP^+^ and NADPH were measured separately based on their differential stabilities in acidic and basic pH. Briefly, the tissue pieces (50 mg) were homogenized with lysis solution (1:1 volume of PBS and 0.2N NaOH with 1% DTAB). For NADP^+^ measurement (acid treatment), 25 µL of 0.4N HCl was added to 50 µL of lysed samples, which were then incubated for 15 min at 60 °C. Simultaneously, 50 µL of the original lysed samples (base treatment) were also incubated for 15 min at 60 °C. After the heat treatment, samples were equilibrated at room temperature for 10 min before neutralizing the acid-treated samples with 25 µL of 0.5 M Trizma^®^ base solution and the base-treated samples with 50 µL of HCl/Trizma solution (1:1 volume of 0.4N HCl and 0.5M Trizma^®^ base). Approximately 100 µL of each acid- and basic-treated samples were added to 96-well white luminometer plates, and the reaction was started by adding 100 µL of NADP/NADPH-GloT™ detection reagent. The reaction was incubated for 1 h before recording the luminescence by a luminometer (SpectraMax, Molecular Devices, Wuxi, China).

### 4.7. GSH and GSSG Assay

The GSH and GSSG levels in cardiac tissues were measured using the GSSG/GSH quantification kit (Dojindo Molecular Technologies Inc., Kumamoto, Japan). Briefly, tissue pieces (50 mg) were homogenized with 1 mL of 5% (*v/v*) 5-sulfosalicylic acid, and the mixture was centrifuged at 8000× *g* for 10 min at 4 °C. Total GSH and oxidized GSH (GSSG) levels in the supernatant were determined according to the manufacturer’s protocol. Reduced GSH levels were calculated based on the concentrations of the total GSH and GSSG that were measured using the following formula: reduced GSH (µmol/L) = total GSH (µmol/L) − 2 × GSSG (µmol/L). The amount of GSH and GSSG was calculated using the standard curve.

### 4.8. Metabolomic Profiling

#### 4.8.1. Sample Preparation

The heart tissue samples were stored at −80 °C until they were used. Tissue pieces (50 mg) were homogenized with 1.2 mL of a solvent mixture (MeOH:H_2_O:CHCl_3_ = 2.5:1:1, v/v) containing 48 µL of 0.5 mg/mL 2-isopropyl-malic acid as an internal standard. The mixture was subsequently shaken at 1200 rpm for 30 min at 37 °C, before being centrifuged at 13,500× *g* for 10 min at 4 °C. About 600 µL of the upper layer was separately collected in another Eppendorf tube, and 200 µL distilled water was added. After vortexing, the mixed solution was centrifuged at 16,000× *g* for 3 min at 4 °C, and 700 µL of the upper layer was moved to another tube. The collected supernatant was concentrated by a centrifugal vaporizer (CVE-200D; Tokyo Rikakikai Inc, Tokyo, Japan) for 25 min, followed by freeze-drying (EYELA FDU-2200; Tokyo Rikakikai Inc, Tokyo, Japan) overnight. Methoxyamine hydrochloride pyridine solution (80 µL, 20 mg/mL) was added to the dried residue. The samples were incubated at 30 °C for 90 min for methoximation. MSTFA (35 µL) was added to 70 µL. The samples were incubated at 37 °C for 30 min for trimethylsilylation, which were used for GC–MS/MS analysis in a split ratio of 1:30. Furthermore, other samples were prepared for methoximation and trimethylsilylation in the 10-fold dilution, which were used for GC–MS/MS analysis in a split ratio of 1:200.

#### 4.8.2. Instrumental Analysis

A GCMS-TQ8040 gas chromatograph–tandem mass spectrometer (Shimadzu, Kyoto, Japan) was used for analysis. A BPX-5 capillary column (30 m × 0.25 mm i.d., film thickness: 0.25 µm, SGE Analytical Science, Ringwood, Victoria, Australia) was used for the chromatographic separation. The column oven temperature was maintained at 60 °C for 2 min and then increased by 15 °C/min to 330 °C, with a final hold for 3 min. Interface and ion source temperatures were 280 °C and 200 °C, respectively. High-purity helium gas was used for carrier gas, and the flow rate was 1.14 mL/min. GC–MS/MS was conducted in the electron ionization positive mode. Ionization mode was at 70 eV. Selected reaction monitoring mode was used for analysis. Metabolites were identified by matching two transitions and their retention time with the Smart Metabolites Database (Shimadzu), performed using a built-in software GCMS solution (version 4.52, Shimadzu). The peak areas of each metabolite were normalized by 2-isopropyl-malic acid. Samples were automatically injected in the split mode and analyzed twice for various analyte intensities. The combination of split ratio and injection volume was 1:30 and 1 µL and 1:200 and 0.5 µL, respectively.

#### 4.8.3. Multivariate Data Analysis

The multivariate data analyses including PCA and heat map were performed using the Web-based software MetaboAnalyst 4.0 (http://www.metaboanalyst.ca) [24]. For the metabolome data map, metabolites of glycolysis, TCA cycle, PPP, glutaminolysis, and polyol pathway were analyzed using a one-way analysis of variance, and fold changes of those metabolites relative to the CT group were calculated.

### 4.9. Statistical Analysis

Statistical analyses were performed using GraphPad Prism 6.0 software (GraphPad Software, CA, USA), and data were presented as the mean ± standard error of the mean. The significance of differences was determined using analysis of variance with post-hoc Tukey’s multiple comparison analysis. Statistical significance was set at *p* < 0.05.

## Supplementary Materials

Supplementary Materials can be found at https://www.mdpi.com/1422-0067/21/22/8858/s1.

## Abbreviations

ATP	Adenosine triphosphate
CO	Carbon monoxide
CS	Cold storage
GC–MS/MS	Gas chromatography/tandem mass spectrometry
GSH	Reduced glutathione
GSSG	Oxidized glutathione
HPG	High-pressure gas
NADPH	Reduced nicotinamide adenine dinucleotide phosphate
O_2_	Oxygen
PCA	Principal component analysis
PCO	Partial pressure of carbon monoxide
PO_2_	Partial pressure of oxygen
PPP	Pentose phosphate pathway
TCA	Tricarboxylic acid
TTC	2,3,5-triphenyltetrazolium chloride
UW	University of Wisconsin
