# Supplementary material for: Cardioprotection via Metabolism for Rat Heart Preservation Using the High-Pressure Gaseous Mixture of Carbon Monoxide and Oxygen
Source: Int J Mol Sci. 2020 Nov 23;21(22):8858. doi: 10.3390/ijms21228858 (PMC7700337; doi:10.3390/ijms21228858)
Supplement: Supplementary file 1 [file ijms-21-08858-s001.zip › Supplementary Materials.docx]

**Supplementary Materials:**

**
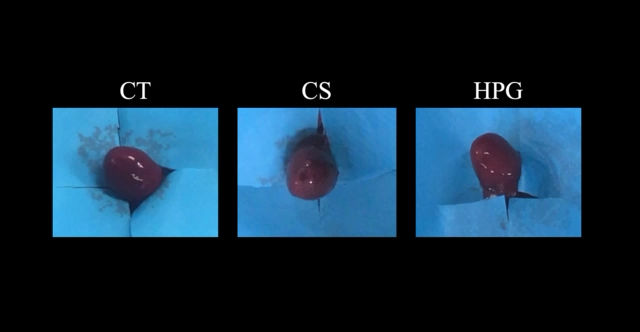
**

**Supplementary Movie.** Heart beating after 60 min after transplantation.

**Supplementary Figure S1.** 3D Principal components analysis (PCA) score plot of metabolome analysis data among CT, CS and HPG groups. PCA was performed using web-based software (MetaboAnalyst 4.0).
